# Characterization of the Structural and Functional Determinants of MANF/CDNF in *Drosophila In Vivo* Model

**DOI:** 10.1371/journal.pone.0073928

**Published:** 2013-09-03

**Authors:** Riitta Lindström, Päivi Lindholm, Jukka Kallijärvi, Li-ying Yu, T. Petteri Piepponen, Urmas Arumäe, Mart Saarma, Tapio I. Heino

**Affiliations:** 1 Department of Biosciences, University of Helsinki, Helsinki, Finland; 2 Institute of Biotechnology, University of Helsinki, Helsinki, Finland; 3 Faculty of Pharmacy, University of Helsinki, Helsinki, Finland; University of Florida, United States of America

## Abstract

Mammalian MANF and CDNF proteins are evolutionarily conserved neurotrophic factors that can protect and repair mammalian dopaminergic neurons *in vivo*. In *Drosophila*, the sole MANF protein (DmManf) is needed for the maintenance of dopaminergic neurites and dopamine levels. Although both secreted and intracellular roles for MANF and CDNF have been demonstrated, very little is known about the molecular mechanism of their action. Here, by using a transgenic rescue approach in the *DmManf* mutant background we show that only full-length MANF containing both the amino-terminal saposin-like and carboxy-terminal SAP-domains can rescue the larval lethality of the *DmManf* mutant. Independent N- or C-terminal domains of MANF, even when co-expressed together, fail to rescue. Deleting the signal peptide or mutating the CXXC motif in the C-terminal domain destroys the activity of full-length DmManf. Positively charged surface amino acids and the C-terminal endoplasmic reticulum retention signal are necessary for rescue of *DmManf* mutant lethality when DmManf is expressed in a restricted pattern. Furthermore, rescue experiments with non-ubiquitous expression reveals functional differences between the C-terminal domain of human MANF and CDNF. Finally, DmManf and its C-terminal domain rescue mammalian sympathetic neurons from toxin-induced apoptosis *in vitro* demonstrating functional similarity of the mammalian and fly proteins. Our study offers further insights into the functional conservation between invertebrate and mammalian MANF/CDNF proteins and reveals the importance of the C-terminal domain for MANF activity *in vivo*.

## Introduction

Neurotrophic factors (NTFs) protect neurons from apoptotic death and promote their regeneration. During development, NTFs regulate neuronal migration, differentiation, maturation, and survival, but also have roles in non-neuronal tissues (reviewed in [1]). The recently discovered MANF/CDNF family of NTFs consists of two paralogues in mammals, MANF (Mesencephalic Astrocyte-derived Neurotrophic Factor; ARMET) [2] and CDNF (Cerebral Dopamine Neurotrophic Factor) [3]. The sole homologue found in invertebrates is more closely related to mammalian MANF than CDNF [4]. Recombinant human MANF (HsMANF) and CDNF (HsCDNF) protect and repair midbrain dopaminergic (DA) neurons in rodent models of Parkinson’s disease *in vivo* [3], [5], [6]. Mammalian MANF can also rescue cortical neurons and cardiomyocytes from ischemia *in vivo* [7], [8]. Importantly, the fly homologue (DmManf) protects DA neurites and maintains DA levels during *Drosophila* development *in vivo* [4]. Based on their DA neuron survival-promoting and neuro-restorative effects, the MANF/CDNF family of proteins has therapeutic potential for treatment of Parkinson’s disease.

**Figure 1:**
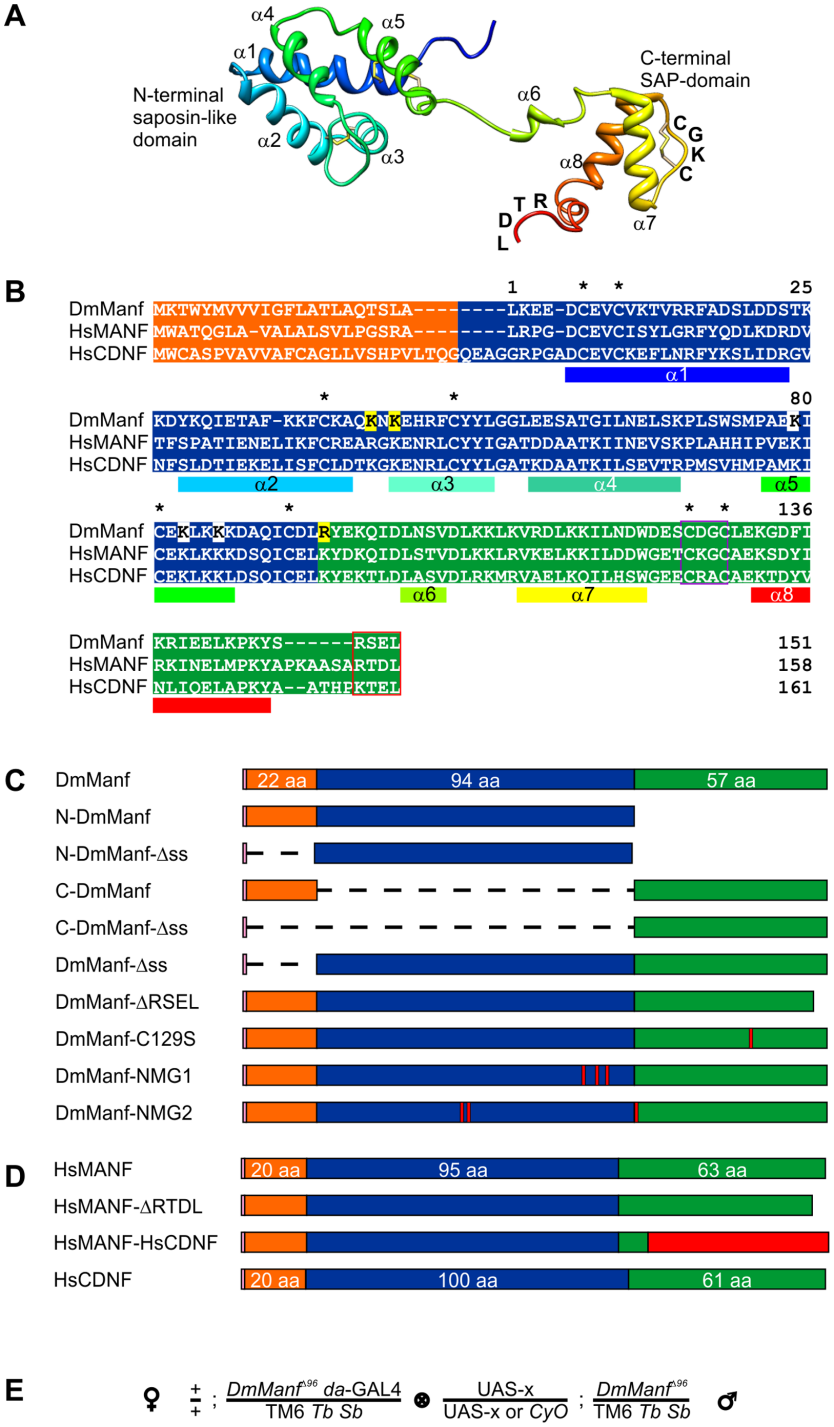
Schematic representation of DmManf and HsMANF constructs and a crossing scheme of rescue experiments. A) 3D structure of HsMANF based on Hellman et al, 2011 [17]. CXXC and RTDL motifs are indicated (image kindly provided by P. Permi). B) Amino acid sequence alignment of DmManf, HsMANF and HsCDNF. Numbering corresponds to DmManf amino acid residues. Signal sequence (ss), N-terminal domain and C-terminal domain are coloured in orange, blue and green, respectively. The eight conserved cysteines are indicated with asterisks. C-terminal CXXC (purple) and RSEL/RTDL/KTEL (red) motifs are rectangled. N-terminal positively charged surface residues mutated in DmManf-NMG1 and DmManf-NMG2 constructs are coloured with white and yellow, respectively. α-helices of HsMANF are marked under the sequence alignment according to colouring in (A). C–D) Schematic presentation of DmManf (C), HsMANF and HsCDNF (D) constructs. Pink represents start codon, orange signal peptide, blue N-terminal domain and green C-terminal domain. Red bars correspond to point mutations. In the HsMANF-HsCDNF construct red corresponds to the HsCDNF sequence. Honeybee melittin was used as a signal peptide in human MANF and CDNF (D) constructs. E) Crossing scheme used in ubiquitous rescue experiments. Rescued homozygous *DmManf^Δ96^* mutant pupae were identified by appearance of *Tb* ^+^ pupae: the expected proportion of fully rescued *DmManf^Δ96^* homozygous mutants is 33% (homozygous UAS-lines) or 17% (heterozygous UAS-lines) by Mendelian inheritance (TM6 *Tb Sb* balancer homozygotes are lethal at early developmental stage). UAS-x, wild type or mutated transgene.

The molecular mechanisms behind the protective properties of MANF/CDNF proteins are still unknown. In addition to the role as a secreted extracellular trophic factor, MANF localizes to the endoplasmic reticulum (ER) and provides a protective function against ER stress *in vitro* [9]–[12]. Consistent with the ER function, both HsMANF and DmManf contain putative ER retention signal sequences (RTDL and RSEL, respectively; Figure 1A–B) in the C-terminal end which resemble the canonical KDEL ER retention signal. The expression of mammalian MANF is up-regulated by chemically induced ER stress *in vitro* [9], [10]. During ER stress, the ER homeostasis is disturbed by accumulation of unfolded proteins leading to activation of the unfolded protein response (UPR; reviewed in [13], [14]). MANF is also shown to bind to a UPR-related ER-resident protein, Glucose-regulated protein 78 (GRP78) in a Ca^2+^-dependent manner *in vitro* [8].

Structurally MANF and CDNF proteins show no amino acid sequence homology to other known families of NTFs, e.g. neurotrophins and glial-cell-line-derived neurotrophic factor (GDNF) family ligands. Human MANF and CDNF consist of two α-helical domains connected by a short flexible linker region (Figure 1A) [15]–[17]. DmManf is expected to adopt a very similar structure because of the high similarity of amino acid sequence and the strict conservation of the spacing between the eight cysteine residues (Figure 1B). The amino (N) -terminal domain (N-MANF and N-CDNF) is structurally homologous to saposin-like proteins (SAPLIPs), a family of lipid-interacting molecules [15]–[19]. The C-terminal domain (C-MANF and C-CDNF) shares the highest structural homology with the SAF-A/B, Acinus and PIAS (SAP) domain of Ku70 protein [17]. Ku70, via the SAP-domain, interacts with a pro-apoptotic protein BCL-2 associated X (Bax) in the cytoplasm and inhibits Bax-mediated apoptotic death of mammalian cells *in vitro* [19]. Similar to Ku70, MANF and cytoplasmic C-MANF protect superior cervical ganglion (SCG) neurons from apoptosis *in vitro* [17]. The C-terminal domain also contains a CXXC motif (127CKGC130 and 126CDGC129 in HsMANF and DmManf, respectively, Figure 1A–B) which forms a disulphide bridge [15], [17] and may participate in oxidation/reduction reactions as a similar CXXC sequence is found in thiol/disulphide oxidoreductases [20].

We have shown in a previous study that zygotic *DmManf* mutant flies die as late first instar larvae and are rescued by ubiquitous expression of transgenic *Drosophila* and human MANF [4]. Here, we used a transgenic approach in the homozygous *DmManf*-deficient mutant fly background to characterize the structural features essential for *in vivo* functioning of the DmManf, HsMANF and HsCDNF proteins. Mutations in *Drosophila* and human MANF and CDNF (Figure 1C–D) were designed based on their known three-dimensional structures [15], [17] and amino acid sequence predictions (Figure 1A–B). Mutations were introduced to *Drosophila* as UAS (upstream activation sequence) -transgenes and expressed ubiquitously by the *da*-GAL4 driver in the homozygous *DmManf* mutant background (Figure 1E). We also studied the conserved role of DmManf in protection of mammalian sympathetic neurons from apoptotic death *in vitro*.

## Results

### Separate N- and C-terminal Domains Fail to Rescue *DmManf* Mutant Lethality

**Figure 2:**
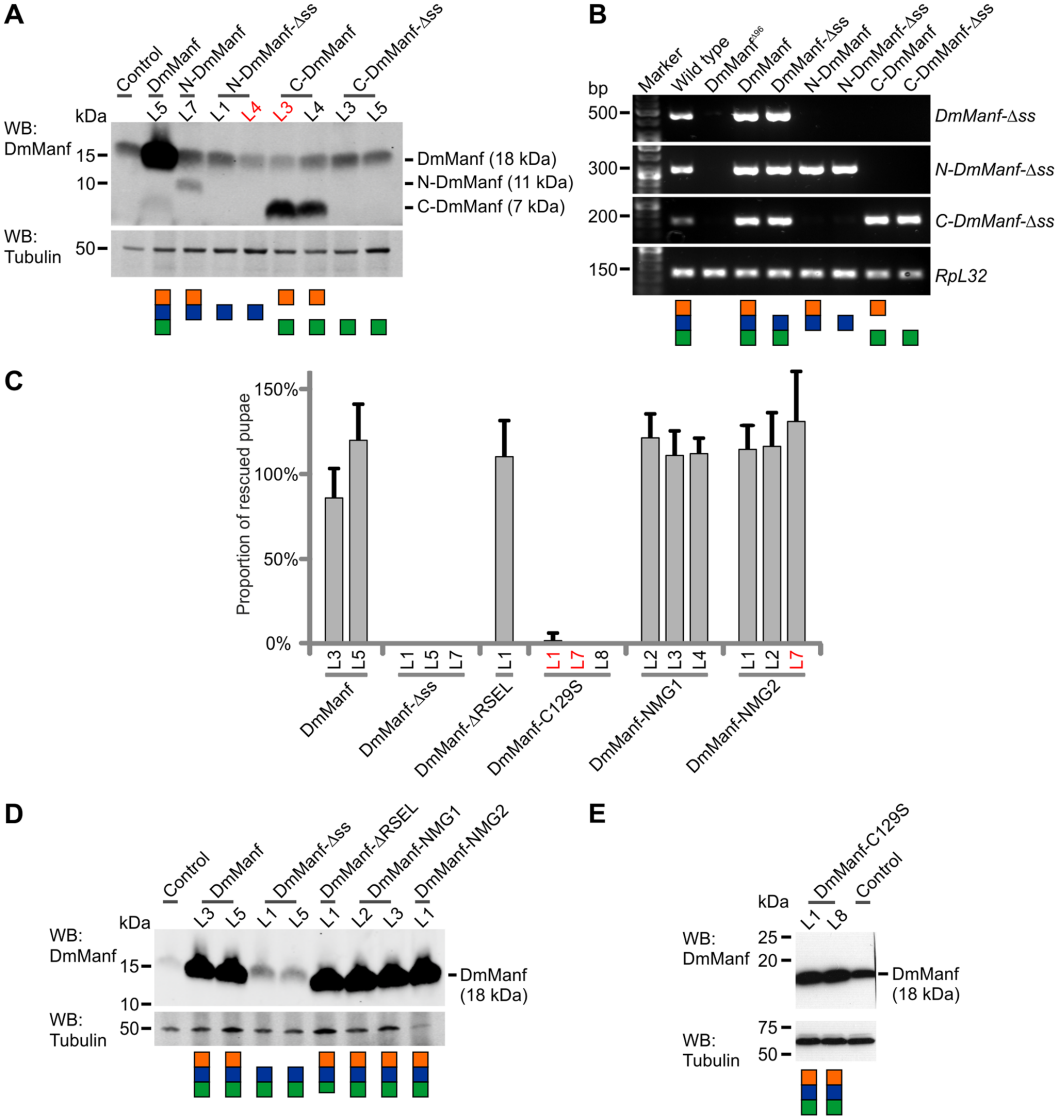
Expression analysis of transgenic DmManf constructs and observed rescue of larval lethality. A) Protein expression of DmManf constructs was verified by Western blotting from 3^rd^ instar larvae. Constructs were ubiquitously expressed by *da*-GAL4 driver in wild type or heterozygous *DmManf* mutant (red typing) background. *da*-GAL4/+ flies were used as control for endogenous DmManf expression. Schematic presentation of each construct coloured according to Figures 1B–C is shown under the blot. Calculated molecular weights of full length proteins, N- and C-terminal domains are presented. L1, L2 etc. correspond to independent transformant lines of each construct. B) *In vivo* transcription from constructs encoding DmManf N- and C-terminal domain was verified by RT-PCR from 1^st^ instar larvae. Constructs were expressed by *da*-GAL4 in homozygous *DmManf* mutant background. DmManf and DmManf-Δss were used as positive controls, homozygous *DmManf* mutant larvae as a negative control. Schematic presentations of each construct are coloured according to Figures 1B–C. C) Rescue of *DmManf^Δ96^* mutant larval lethality by transgenic wild type or mutated constructs of DmManf ubiquitously expressed by *da*-GAL4. L1, L2 etc. correspond to independent transformant lines of each construct. Average ± SD. D) Western blot analysis of protein expression of DmManf constructs with similar to size to endogenous DmManf (DmManf, DmManf-Δss, DmManf-ΔRSEL, DmManf-NMG1, and DmManf-NMG2). The constructs were ubiquitously expressed by *da*-GAL4 in wild type background. Expression from a transgenic construct is detected as upregulated protein levels in comparison to endogenous DmManf levels in control flies (*da*-GAL4/+). L1, L2 etc. correspond to independent transformant lines of constructs. E) Expression analysis of the DmManf-C129S construct in embryos by Western blotting. Constructs were expressed by *da*-GAL4 in wild type background. Transgenic expression is detected as upregulation of DmManf expression compared to *da*-GAL4 flies (control). In Western blot analyses, alpha-tubulin was used as loading control.

To explore the function of the two domains of MANF (Figure 1A) we asked whether either of the domains, as an independent unit, could rescue *DmManf* mutant lethality *in vivo*. First, the N-terminal (residues 1–94) or C-terminal domain (residues 95–151) of mature DmManf was expressed ubiquitously by *da*-GAL4 either with (N-DmManf, C-DmManf) or without a signal peptide (residues ss2–ss22; N-DmManf-Δss, C-DmManf-Δss; Figure 1B–C) in the *DmManf* mutant background. In contrast to full-length DmManf, none of these constructs could rescue the early larval lethality (Table 1). The expression of N- and C-terminal UAS-transgenes was verified by overexpressing the constructs by ubiquitous *da*-GAL4 in wild type background. Expression of N-DmManf and C-DmManf with a signal peptide was detected from larval lysates by Western blotting (Figure 2A). Unfortunately, the protein expression level of N-DmManf-Δss and C-DmManf-Δss constructs which lack the signal peptide was below the detection limit. Reverse transcription polymerase chain reaction (RT-PCR) from 1^st^ instar larvae indicated that the mRNAs were expressed *in vivo* (Figure 2B) suggesting that either the translation or the stability of N-DmManf-Δss and C-DmManf-Δss proteins were compromised.

**Table 1:** Number of heterozygous pupae in rescue experiments with N- and C-terminal constructs of DmManf.

Construct and insertion	Number of balancedpupae counted
N-DmManf L7	1314
N-DmManf L1	1213
N-DmManf-Δss L1	983
N-DmManf-Δss L3.2	1172
N-DmManf-Δss L4	1409
C-DmManf L3	1799
C-DmManf L4	1614
C-DmManf-Δss L3	2051
C-DmManf-Δss L5	1894
N-DmManf L7+C-DmManf L3	661
N-DmManf L7+C-DmManf L4	910
N-DmManf L7+C-DmManf-Δss L3	597
N-DmManf L7+C-DmManf-Δss L5	977
N-DmManf-Δss L1+C-DmManf L3	1152
N-DmManf-Δss L1+C-DmManf L4	910
N-DmManf-Δss L1+C-DmManf-Δss L3	781
N-DmManf-Δss L1+C-DmManf-Δss L5	508

No homozygous *DmManf^Δ96^* mutant pupae were found in any of the rescue experiments listed here. L:s correspond to independent insertions.

Next we studied whether the *DmManf* mutant lethality can be rescued by the N- and C-terminal domains of DmManf expressed together as two separate transgenes by ubiquitous *da*-GAL4 driver (Figure S1A). Interestingly, the two co-expressed independent domains also failed to complement the loss of endogenous DmManf (Table 1) suggesting that intact DmManf protein containing both domains is needed for *in vivo* activity.

Interestingly, when wild type DmManf was overexpressed, a second very faint band corresponding to the size of C-DmManf was detected in addition to the expected band of approximately 18 kDa (Figure 2A). This suggests that abundantly expressed DmManf is partially degraded *in vivo* releasing the C-terminal domain.

### ER Entry but not ER Retrieval is Essential for DmManf Function *in vivo*

Both mammalian and *Drosophila* MANF localize to the ER and are also secreted [4], [9]–[12], [21]. MANF has a signal peptide in the N-terminus that directs newly synthesized protein into the ER (Figure 1B). Since MANF has both extracellular neuro-protective [4], [5] and intracellular cyto-protective functions [17], we wanted to test whether entry into the ER and subsequent secretion of DmManf is crucial for its functionality. Therefore, we designed a DmManf transgene with a deletion of the signal sequence (aa ss2–ss22; DmManf-Δss, Figure 1C). Ubiquitous expression of DmManf-Δss could not rescue the early larval lethality of *DmManf* mutants (Figure 2C) suggesting that DmManf entry into the secretory pathway via the ER is necessary for its function during development. Protein expression from DmManf-Δss construct was confirmed from larvae by Western blot analysis (Figure 2D). Interestingly, the DmManf-Δss showed lower protein expression levels than wild type DmManf although the mRNA was expressed *in vivo* (Figure 2B). This suggests that DmManf-Δss which is not targeted to ER and, instead, processed in the cytoplasm is either unstable or its translation is compromised. Use of two independent insertions of the DmManf-Δss transgene and two copies of *da*-GAL4 driver did not notably increase the expression level (Figure S1B) and failed to rescue *DmManf* mutant lethality (Table 2).

**Table 2:** Number of heterozygous pupae in rescue experiments with different expression levels of the DmManf-Δss construct.

Construct and insertion	*da*-GAL4 dosage	Number of balanced pupae counted
DmManf-Δss L1	1x	1031
DmManf-Δss L5	1x	1065
DmManf-Δss L1+DmManf-Δss L5	1x	1360
DmManf-Δss L5+DmManf-Δss L1	1x	1455
DmManf-Δss L1+DmManf-Δss L5	2x	808
DmManf-Δss L5+DmManf-Δss L1	2x	479

No homozygous *DmManf^Δ96^* mutant pupae were found in any of the rescue experiments listed here. L:s correspond to independent insertions.

**Figure 3:**
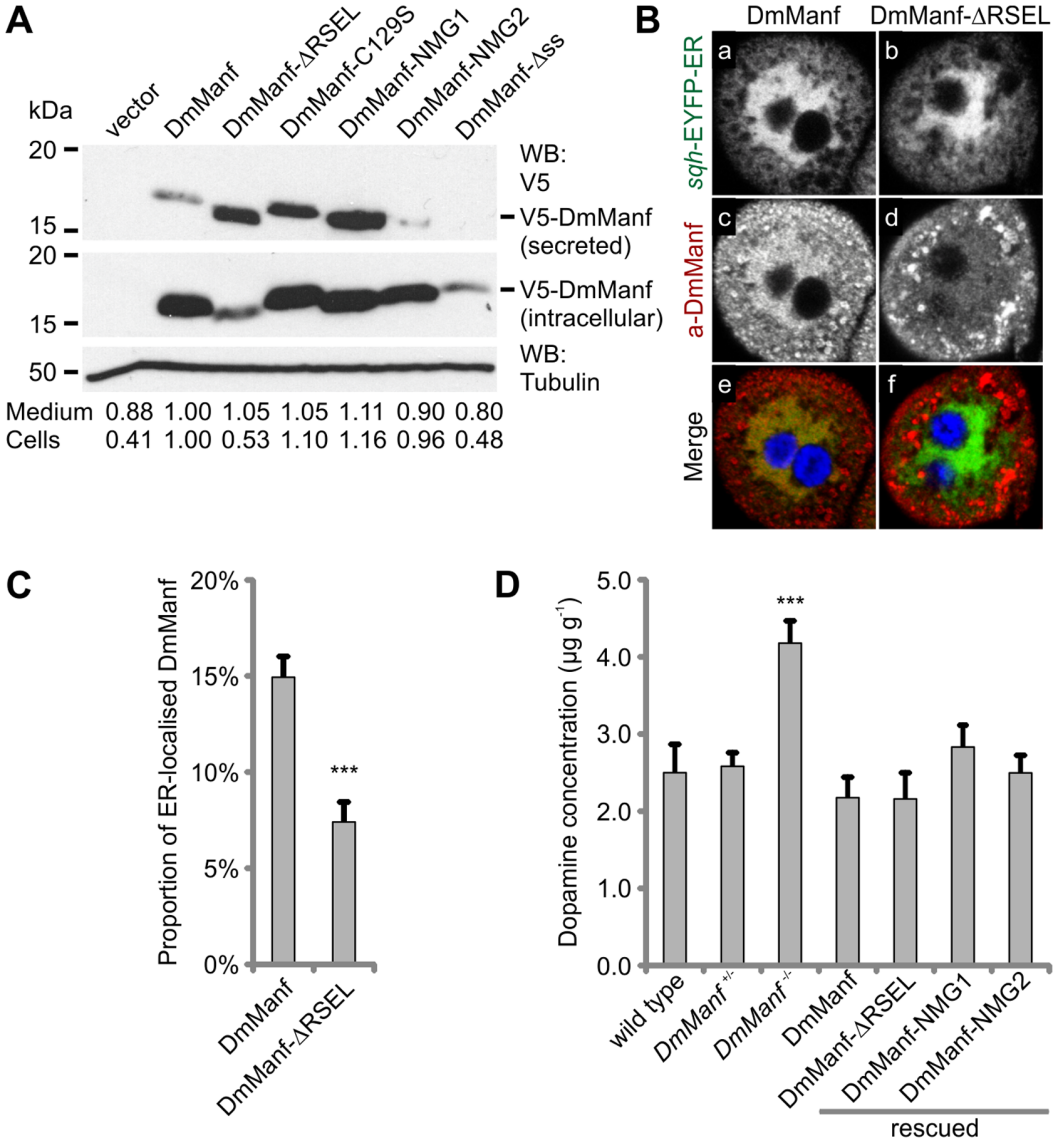
Deletion of RSEL sequence affects secretion and intracellular localisation of DmManf. A) Western blot analysis of cellular and secreted DmManf *in vitro*. Schneider 2 cells were transiently transfected with wild type and mutated V5-DmManf-pMT constructs. DmManf protein levels in cell lysates and conditioned medium were estimated according to Figure S1G. Band intensities were normalised to DmManf. Alpha-tubulin was used as a loading control. B) DmManf-ΔRSEL driven by ubiquitous *da*-GAL4 (b, d and f) showed decreased endoplasmic reticulum (ER) localisation compared to wild type DmManf (a, c and e) in 3^rd^ instar larval garland cells of homozygous *DmManf^Δ96^* mutants. Yellow (e and f) represents the co-localisation of the ER marker (*sqh*-EYFP-ER; green; a and b) EYFP and a-DmManf (red; c and d). Nuclei were stained with DAPI (blue; e and f). C) Quantification of (B). Deletion of the putative ER retention signal (RSEL) significantly decreased the proportion of ER localised DmManf of total cellular DmManf in 3^rd^ larval garland cells compared to the wild type construct. Average ± SEM, *n* = 13. ***, *P*<0.001, Student’s *t*-test. D) *DmManf* mutant (−/−) larvae (*n* = 13) showed significant increase in dopamine concentration in comparison to wild type (*n* = 8). Ubiquitous expression of DmManf (*n* = 11), DmManf-ΔRSEL (*n* = 10), DmManf-NMG1 (*n* = 8) or DmManf-NMG2 (*n* = 10) by *da*-GAL4 in homozygous *DmManf* mutant background rescued dopamine concentration to the wild type level. ***, *P*<0.001 versus wild type, Student’s *t*-test.

We studied the secretion of DmManf-Δss *in vitro* by transiently transfecting *Drosophila* Schneider 2 (S2) and mammalian Chinese hamster ovary (CHO) cells with V5-DmManf-Δss-pMT and DmManf-Δss-pCR3.1 constructs encoding V5-DmManf-Δss and DmManf-Δss, respectively. In S2 cell transfections V5-tagged constructs were used to distinguish endogenously produced DmManf protein and the protein expressed from transfected plasmids. After incubation for 72 h, V5-DmManf-Δss and DmManf-Δss were detected in cell extracts but not in the medium of S2 or CHO cells by Western blotting (Figure 3A and Figure S1C) showing that DmManf-Δss was not secreted. We also used *salm*-GAL4 to express DmManf and DmManf-Δss in 3^rd^ instar larval wing discs and detected up-regulation of DmManf immunoreactivity specifically in the GAL4 expression pattern (indicated by nuclear GFP; Figure S1D). This *in vivo* analysis further indicated a lack of secreted DmManf-Δss since it showed more strict expression in the GFP-expressing cells whereas wild type DmManf also localized next to them (white arrows in Figure S1Dh–i).

We also created a DmManf mutant transgene with deletion of the putative ER retention signal RSEL (DmManf-ΔRSEL; Figure 1B–C) to study whether ER retention of DmManf is crucial for functionality. Ubiquitously expressed DmManf-ΔRSEL rescued the larval lethality of *DmManf* mutants to adulthood (Figure 2C and Figure S1E). The localization of DmManf inside the ER (Figure 3B) in 3^rd^ instar larval garland cells of homozygous *DmManf* mutants was significantly decreased when the putative ER retention signal was deleted (Figure 3B–C). Thus, the retention of abundantly expressed DmManf in the ER is not essential for fly viability.

### CXXC Motif in the C-terminal Domain is Crucial for *in vivo* Functionality of DmManf

The crystal and solution NMR structures of mature HsMANF and HsCDNF revealed that the C-terminal domain contains a CXXC motif (residues 127–130) forming a disulphide bridge (Figure 1A–B) [15]–[17]. We created a DmManf transgene with a point mutation that replaces cysteine-129 with serine (C129S; Figure 1C), a mutation that destroys the CXXC motif and prevents the formation of the C-terminal disulphide bridge (Figure 1A). Interestingly, this full-length DmManf transgene with a mutant C-terminal motif (DmManf-C129S) did not rescue the *DmManf* mutant lethality (Figure 2C). Only one of the three tested insertions resulted in very few rescued pupae (DmManf-C129S L1 in Figure 2C). The expression of DmManf-C129S in *Drosophila* under ubiquitous *da*-GAL4 driver was verified by Western blotting (Figure 2E). The secretion of V5-DmManf-C129S was not compromised in comparison to V5-DmManf in transiently transfected S2 cells analyzed by Western blotting (Figure 3A).

### Positive Charge on the Surface of the N-terminal Domain of DmManf is not Essential for Fly Viability

The N-terminal domain of MANF is characterized by a globular saposin-fold of five α-helices stabilized by three disulphide bridges [15]–[17]. Two groups of positively charged surface amino acids were identified in the N-terminal domain of HsMANF which could interact with negatively charged phospholipids in the cell membrane [15]. To disrupt the putative lipid interaction, we designed two constructs in which selected positively charged lysines and arginines of one of the surface amino acid groups were replaced with neutral alanines: DmManf-NMG1 (N-terminal Mutant Group 1) containing amino acid changes K79A, K83A and K86A, and DmManf-NMG2 with mutations K43A, K45A and R95A (Figure 1C and Figure 2D). In the *DmManf* mutant background, neither of these mutation groups abolished the functionality of DmManf when ubiquitously expressed, i.e. both of the constructs rescued larval lethality of the *DmManf* mutants (Figure 2C).

### Increased Dopamine Levels of *DmManf* Mutants are Normalized in Rescued Larvae

The lack of endogenous DmManf leads to the loss of dopaminergic neurites in larval central nervous system (CNS) [4]. Abolishment of both maternal and zygotic DmManf shows a substantial decrease in dopamine levels at the end of embryogenesis [4]. We wanted to characterize the dopaminergic system of rescued larvae and compare it with that of the *DmManf* mutant. First, we tried to analyze the DA neurites of larval CNS by staining with an antibody against tyrosine hydroxylase (TH), an essential enzyme of DA synthesis and a marker for DA neurons. Unfortunately, this immunohistochemical approach was not sensitive enough. Instead, we measured the dopamine levels of *DmManf* mutants and rescued larvae to address the phenotypes associated with the dopaminergic system. We collected larvae at the latest stage when the lethality of homozygous *DmManf* mutants occurs. Homozygous *DmManf* mutants showed a significant increase in dopamine levels in total larval lysates compared to wild type larvae (Figure 3D). All constructs driven by *da*-GAL4 that were able to rescue larval lethality (DmManf, DmManf-ΔRSEL, DmManf-NMG1 and DmManf-NMG2) also normalized larval dopamine to the wild type level (Figure 3D).

### Restricted Expression of Mutated Constructs Reveals their Effects on DmManf Functionality

**Figure 4:**
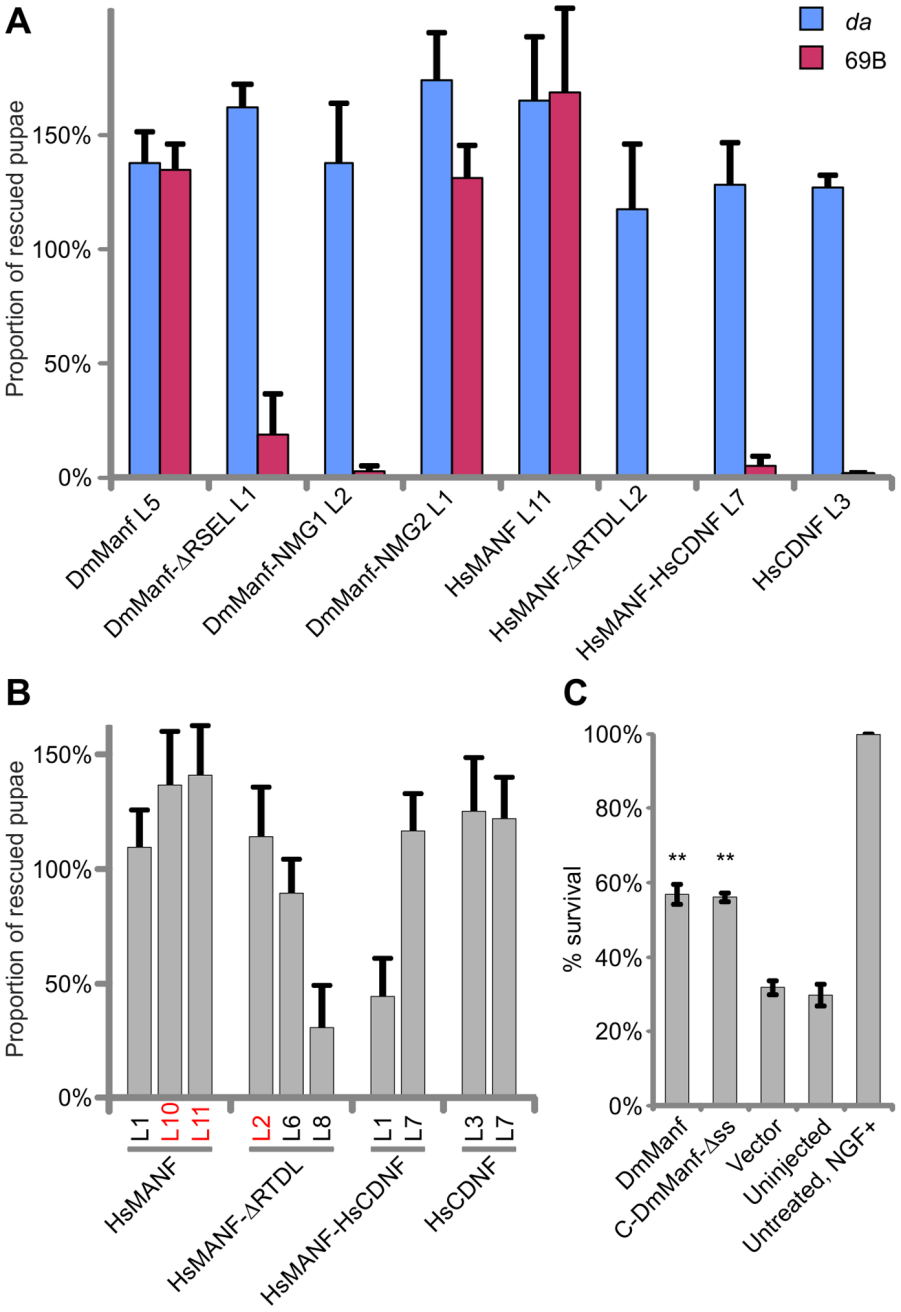
DmManf shares conserved functions with human MANF and CDNF. A) Comparison of the rescue efficacy of *DmManf^Δ96^* mutant lethality between ubiquitous *da*-GAL4 and non-ubiquitous 69B-GAL4 drivers with selected transgenic DmManf, HsMANF, and HsCDNF constructs. L1, L2 etc. correspond to independent transformant lines of each construct. Average ± SD. B) Rescue of *DmManf^Δ96^* mutant larval lethality by transgenic wild type or mutated constructs of HsMANF and HsCDNF ubiquitously expressed by *da*-GAL4. L1, L2 etc. correspond to independent transformant lines of each construct. Average ± SD. C) Full-length DmManf (*n* = 5) and an independent C-terminal domain (C-DmManf-Δss, *n* = 2) rescued superior cervical ganglion neurons from apoptotic deathsimilar to HsMANF and C-HsMANF [17]. The NGF-maintained neurons were microinjected with the indicated expression plasmids or empty vector (negative control) and treated with etoposide (30 µM). The injected neurons were identified by the EGFP expression of the co-injected EGFP plasmid. Living neurons were counted three days later and presented as a proportion of the original injected neurons. The NGF-maintained untreated neurons show that no cell death occurs without etoposide treatment. Average ± SEM. **P<0.01 versus control plasmid (Vector) by one-way ANOVA and *post hoc* Dunnett’s test.

When expression of DmManf constructs is driven by *da*-GAL4, proteins encoded by transgenes are available abundantly and ubiquitously in all tissues. This may mask the effects of mutations, especially if they affect the proportion of secreted MANF, intracellular dynamics or extracellular distribution of the protein. To assess this, we expressed DmManf, DmManf-ΔRSEL, DmManf-NMG1 and DmManf-NMG2 transgenes by a non-ubiquitous 69B-GAL4 which is not expressed in muscles, fat body or gastric caeca. Expression in the CNS and cuticle is clearly decreased compared to *da*-GAL4. Despite the restricted expression pattern, the wild type DmManf construct driven by 69B-GAL4 resulted in full rescue of the *DmManf* mutant (Figure 4A and Figure S1E) as previously shown [4]. The overall GAL4 expression level was slightly weaker in 69B-GAL4 than *da*-GAL4 analyzed by Western blotting (Figure S1F). However, with both drivers the expression of ectopic DmManf was clearly higher than the endogenous level of DmManf (Figure S1F).

Deletion of the ER retention signal (DmManf-ΔRSEL) clearly reduced the proportion of rescued pupae with 69B-GAL4 driver compared to ubiquitous *da*-GAL4 driver (Figure 4A). We analyzed whether the ΔRSEL mutation affected the secretion of DmManf by transiently transfecting S2 cells with V5-DmManf-pMT and V5-DmManf-ΔRSEL-pMT plasmids. In comparison to V5-DmManf, the level of cellular V5-DmManf-ΔRSEL protein was clearly decreased while the level of secreted V5-DmManf-ΔRSEL in the medium was at least as high as the level of secreted V5-DmManf, indicating that the proportion of secreted DmManf was increased (Figure 3A). These data suggest that ER retention is important for correct DmManf function when protein availability is spatially and/or temporally restricted.

When expressed by 69B-GAL4 driver, DmManf-NMG2 fully rescued the larval lethality of *DmManf* mutants similar to the wild type DmManf construct (Figure 4A). In contrast, restricted expression of the DmManf-NMG1 construct by 69B-GAL4 lead to almost complete loss of rescued *DmManf* mutant pupae (Figure 4A). Similar amounts of secreted and cellular V5-DmManf were detected from S2 cells transiently transfected with V5-DmManf-pMT, V5-DmManf-NMG1-pMT and V5-DmManf-NMG2-pMT constructs (Figure 3A). These data demonstrate that the neutralization of positively charged surface amino acids K79, K83 and K86 (DmManf-NMG1) but not K43, K45 and R95 (DmManf-NMG2) affect other molecular properties of DmManf than the secretory efficacy.

### DmManf is Functionally more Related to Human MANF than CDNF

In our previous studies transgenic human CDNF (HsCDNF-6N9C; Figure S2A) did not rescue the *DmManf* mutant lethality [4]. Because the HsCDNF-6N9C construct used in the previous rescue experiments encoded HsCDNF with some extra N- and C-terminal amino acid residues (which were introduced into the construct during cloning) we decided to remove these additional residues and generate a new HsCDNF transgene (Figure 1D). To our surprise, this HsCDNF transgene expressed by *da*-GAL4 could, as efficiently as HsMANF, rescue the *DmManf* mutant lethality to adulthood (Figure 4B and Figure S1E). The extra N-terminal amino acids were likely responsible for deficient functionality of the HsCDNF-6N9C construct (Figure S2A–C).

To study the functionality of the independent N- and C-terminal domains of human MANF and CDNF in the *Drosophila in vivo* model, we designed mutated human MANF (N-HsMANF, C-HsMANF, C-HsMANF-Δss; Figure S3A) and CDNF (N-HsCDNF, C-HsCDNF, C-HsCDNF-Δss; Figure S2A) constructs. In line with the corresponding DmManf constructs (see above), these constructs could not rescue the larval lethality of *DmManf* mutants (Table 3). While only N-HsCDNF was detected from larval lysates by Western blotting (Figures S3B–C), mRNA expression was verified by RT-PCR from adult fly lysates of all of these constructs (Figures S3D–E).

**Table 3:** Number of heterozygous pupae in rescue experiments with N- and C-terminal constructs of HsMANF and HsCDNF.

Construct and insertion	Number of balanced pupae counted
N-HsMANF L8	1889
C-HsMANF L2	1246
C-HsMANF L9	1469
C-HsMANF-Δss L2	1510
C-HsMANF-Δss L6	1508
N-HsCDNF L3	1325
N-HsCDNF L4	1143
C-HsCDNF L4.1	1482
C-HsCDNF L5	1608
C-HsCDNF-Δss L5	1266
C-HsCDNF-Δss L7	1274

No homozygous *DmManf^Δ96^* mutant pupae were found in any of the rescue experiments listed here. L:s correspond to independent insertions.

We used non-ubiquitous expression of the HsMANF and HsCDNF transgenes to rescue *DmManf* mutant lethality by 69B-GAL4 driver. With a restricted expression pattern, HsMANF could fully rescue *DmManf* mutant lethality to adulthood, while HsCDNF showed clearly decreased amount of rescued pupae and no emerging adults (Figure 4A and Figure S1E). This suggests that human MANF and CDNF have diverged during evolution. We also asked whether the C-terminal domain of HsCDNF could complement the function of C-HsMANF by replacing the C-terminal domain of HsMANF (residues 105–158) with that of HsCDNF (residues 101–164; HsMANF-HsCDNF; Figure 1D). Ubiquitous expression of this construct rescued *DmManf* mutant lethality to adulthood (Figure 4B and Figure S1E). However, the proportion of rescued pupae with restricted expression of HsMANF-HsCDNF was clearly decreased compared to HsMANF, but closely resembled that of HsCDNF (Figure 4A). This demonstrated that functional properties of the C-terminal domain of HsCDNF differ from HsMANF and DmManf. To test this hypothesis we designed a construct with the N-terminal domain of HsCDNF (residues 1–100) and the C-terminal domain of HsMANF (residues 96–158), but failed to obtain transgenic flies.

We found that ubiquitous but not restricted expression of HsMANF-ΔRTDL, an HsMANF transgene with deletion of the ER retention signal RTDL (Figure 1D), could rescue *DmManf* mutant lethality (Figure 4B and Figure S1E). Correspondingly, the DmManf construct (DmManf-ΔRSEL) expressed in a restricted pattern decreased the amount of rescued *DmManf* mutant pupae. This further supports the importance of ER retained MANF during fly development.

### DmManf Rescues Mouse SCG Neurons from Apoptosis *in vitro*

After studying the functionality of mammalian MANF/CDNF in the *Drosophila* system we addressed the opposite, i.e. can DmManf rescue the mammalian cells. We used cultured apoptotic SCG neurons overexpressing the MANF constructs as this is currently the only reliable mammalian *in vitro* bioassay for MANF/CDNF. At neonatal stage the SCG neurons require nerve growth factor (NGF) for survival but are also sensitive to other apoptotic stimuli, thus being an excellent model to test the survival-promoting compounds. Our recent studies have shown that a plasmid encoding HsMANF or purified recombinant HsMANF protein protects mouse SCG neurons from apoptosis when microinjected into the neuronal nucleus or cytoplasm, respectively [17]. Independent cytoplasmic C-HsMANF-Δss also shows survival promoting activity in SCG neurons. Interestingly, overexpressed HsMANF protects the neurons against etoposide treatment, but only marginally against NGF deprivation [17]. We microinjected the NGF-maintained SCG neurons derived from neonatal (P0) mouse with DmManf-pCR3.1 and C-DmManf-Δss-pCR3.1 plasmids and treated neurons with etoposide to induce apoptosis. Importantly, both full-length DmManf with the endogenous fly signal peptide and the intracellular C-DmManf-Δss were able to rescue mouse SCG neurons from apoptosis (Figure 4C), similar to HsMANF *in vitro* [17]. To eliminate the possibility of intracellular DmManf leakage from apoptotic cells into the medium, we transfected etoposide-treated mammalian CHO cells with DmManf-pCR3.1 and DmManf-Δss-pCR3.1 constructs. In contrast to DmManf, no DmManf-Δss protein was detected in the medium sample by Western blot analysis (Figure S1C).

## Discussion

A neuro-protective function for MANF and CDNF has been demonstrated both *in vitro* [2], [17] and *in vivo* [3]–[6]. Since very little is known about the mechanism of MANF and CDNF action, we used a transgenic approach in the *Drosophila Manf* mutant model to characterize the structural features of MANF/CDNF proteins needed for activity *in vivo*. We first used ubiquitous expression of transgenes and based on obtained results, used non-ubiquitous expression of selected transgenes. While ubiquitous abundant expression should provide sufficient MANF concentration for all cells it could mask some effects of the designed mutations affecting intracellular dynamics, extracellular distribution, or ratio of intra- and extracellular MANF levels. Therefore restricted expression (in which the transgenic wild type DmManf construct fully rescues) may reveal the effects of mutations more efficiently.

Previous studies have demonstrated that MANF is an ER resident protein upregulated by ER stress [9]–[12], [21]. Loss of the relatively weak ER retention signal RTDL leads to increased secretion of MANF [8], [22]. In this study we showed that deletion of the C-terminal RSEL sequence also increased secretion of DmManf and significantly decreased its localization to the ER. We found that ER retention of MANF was not absolutely necessary for MANF function *in vivo* since ubiquitously expressed DmManf-ΔRSEL was able to rescue *DmManf* mutant lethality. However, the restricted expression of DmManf-ΔRSEL or HsMANF-ΔRTDL did not fully rescue *DmManf* mutant lethality. This suggests that limited availability of MANF inside the cell compromises the intracellular functions of MANF.

Our study showed that signal sequence-mediated entry to the secretory pathway via the ER is crucial for the proper function of MANF since DmManf-Δss was unable to rescue *DmManf* mutant lethality. Furthermore, the MANF protein synthesized in the cytoplasm seemed to be unstable. In future studies, it would be important to address whether the loss of the ER resident or secreted pool of MANF was crucial for the functional failure of the DmManf-Δss mutant.

Interestingly, we found that the CXXC motif in the C-terminal domain is crucial for DmManf function *in vivo* since the DmManf-C129S mutant could not complement for the loss of the endogenous DmManf protein. One possibility is that MANF could relieve ER stress by helping proteins to fold properly [1] by facilitating intracellular disulphide bond formation via the C-terminal CXXC motif and thus functionally resemble thiol/oxidoreductases [1], [20]. However, no oxidoreductase activity of MANF was found *in vitro* [9]. Mutation of cysteine-129 abolishes the CXXC disulfide bond which may result in an inactive conformation of the protein. The thiol groups could also perform other essential functions, such as redox signaling or metal ion binding that might be defected in the DmManf-C129S mutant. Additionally, the MANF CXXC motif is within the EF-hand Ca^2+^ binding domain (FlyBase ID FBgn0027095, [23]). A C129S mutation putatively affecting the folding or conformation of the C-terminal domain might lead to loss of Ca^2+^-dependent GRP78 binding to MANF.

In contrast to zygotic and maternal *DmManf* mutant embryos [4], the homozygous zygotic *DmManf* mutant larvae showed increased dopamine levels. Microarray analysis [12] revealed in both embryonic and larval *DmManf* mutants upregulation of TH transcript expressed specifically in the hypoderm [24]. Hypodermal cells give rise to cuticle where DA derivatives are used as cross-linkers. In zygotic and maternal *DmManf* mutant embryos with decreased DA levels the total lack of DmManf could lead to deficient DA synthesis despite increased TH transcript levels. While the zygotic mutant larvae still possess a pool of maternal DmManf, increased DA synthesis might remain with an increased rate due to the elevated amounts of TH until the maternal pool of DmManf has been depleted.

DmManf also efficiently protected apoptotic mammalian neurons. Although the *in vitro* SCG neuron model and the *in vivo* fly rescue models are not directly comparable, this result shows that the basic neuro-protective function of MANF has persisted during evolution. However, the protective mechanisms in these two models may significantly differ, as C-DmManf-Δss protected SCG neurons but was ineffective in rescuing the mutant fly phenotype. Moreover, the MANF/CDNF N- or C-terminal domains alone, even if expressed together within the same cells, could not rescue the larval lethality of *DmManf* mutants demonstrating that an intact full-length protein is crucial for fly viability.

In conclusion, our study provides the first *in vivo* demonstration of structural and functional determinants of the MANF/CDNF protein family.

## Materials and Methods

### Fly Stocks and Maintenance

Flies were maintained at 25°C on malt-based media. The fly stocks used were: *w* ^-^, UAS-*DmManf^133^* (insertion L3), UAS-*DmManf^135^* (insertion L5), UAS-HsCDNF-6N9C, and *DmManf^Δ96^*/TM6 *Tb Sb* [4]; UAS-lacZ [25]; UAS-GFP.nls #4775 [26], *sqh*-EYFP-ER #7195 [27], *da*-GAL4 #5460 [28], 69B-GAL4 #1744 [25], *tub*-GAL4 #5138 [29], and *salm*-GAL4 #5818 [30] from Bloomington *Drosophila* Stock Center.

### Generation of Transgenic Flies

Mutations were generated by PCR mutagenesis using Phusion® Hot Start High-Fidelity DNA polymerase (Finnzymes) and subcloned into the pUAST vector [25] (Table S1). Most mutations were created on templates in pBlueScript SK or pCR3.1 (Invitrogen) vectors (Table S1). The remaining constructs or templates were created according to the following (numbers correspond to Table S1, primers are listed in Table S2): (1) **DmMANF-K95A-Bluescript** was created by inverse PCR mutagenesis with primers DmMANF_R95A.fwd and DmMANF-Nterm.rev, DmMANF-BlueScript [4] was used as a template; (2) **HsMANF-pCR3.1** – HsMANF with honeybee melittin secretion signal [4] was subcloned as an *Eco*RI-*Xho*I fragment from the pUAST vector into pCR3.1. To create FL-HsMANF without extra C-terminal residues, a stop codon was introduced by PCR; (3) **HsMANF-HsCDNF-pCR3.1** – a C-terminal fragment of HsCDNF with *Bpu*10I and *Xho*I sites was generated by PCR. FL-HsMANF in pCR3.1 was restricted with *Bpu*10I and *Xho*I and the C-terminus of HsMANF was replaced with C-HsCDNF; (4) **NaeI-HsCDNF-pCR-II** – a PCR fragment of HsCDNF with an N-terminal NaeI site and a C-terminal stop codon was created and ligated into the pCRII (Invitrogen) vector. The cDNA encoding CDNF in HsCDNF-6N9C-pUAST [4] was replaced with a *Nae*I- HsCDNF- *Xho*I fragment from pCRII to create HsCDNF-pUAST; (5) **HsCDNF-Bluescript** was cloned as a *Not*I-*Xho*I fragment from a PCR product in which HsCDNF-pUAST was used as a template with the primers NotI-CDNF-pUAST.fwd and CDNF-pUAST-XhoI; (6) **C-HsCDNF-Δss-pUAST** was cloned as a *Bgl*II-*Xho*I fragment from a PCR product of NaeI-HsCDNF-pCR-II with the primers BglII-M-HsCDNF-Cterm.fwd and HsCDNF-TOPO-XhoI.rev; (7) **HsCDNF-6N-BlueScript** was subcloned as a *Not*I-*Bgl*II fragment from HsCDNF-6N9C-delStop-BlueScript to HsCDNF-BlueScript; (8) **HsCDNF-6N9C-del(stop)-Bluescript** was cloned as a *Not*I-*Xba*I fragment from a PCR product in which HsCDNF-6N9C-pUAST [4] was used as a template with the primers NotI_CDNF_old_pUAST.fwd and CDNF_old_pUAST_XbaI.rev. The template vector DmManf in pBlueScript was kindly provided by M. Palgi [4]. All constructs were verified by sequencing. Honeybee melittin was used as a signal peptide in human MANF and CDNF. Transgenesis was performed by Genetic Services, Inc. Selected insertions in the 2^nd^ chromosome were combined with *DmManf^Δ96^* mutations balanced against TM6 *Tb Sb*.

### Rescue Experiments

Five GAL4 *DmManf^Δ96^*/TM6 *Tb Sb* females were crossed to 2–3 UAS-x(/*CyO*); *DmManf^Δ96^*/TM6 *Tb Sb* males. Five parallel crosses were done and transferred twice to new vials every 2–3 days. Normal (*Tb* ^+^) and short (*Tb* ^-^) pupae were counted 9–10 days after egg laying (AEL). The amount of normal pupae was divided by the amount of all pupae and the ratio normalized by 0.33 (homozygous UAS lines) or 0.17 (heterozygous UAS lines). For quantification of rescued adults, amounts of rescued pupae (*Tb* ^+^) and adults (*Sb* ^+^) were counted 10 days AEL and daily 10–14 AEL, respectively. Emerged adults are presented as proportion of rescued pupae.

### Immunoblotting

Larvae were homogenized in 300 µl lysis buffer (20 mM Tris-Cl pH 7.4, 150 mM NaCl, 1% Triton X-100, 1 mM EDTA) supplemented with Complete proteinase inhibitor tablets (Roche). Cultured cells were washed once with PBS and lysed in membrane lysis buffer (TBS, 1% Triton X-100, 20 mM NaF, 1 mM EDTA, pH 7.5) containing Complete proteinase inhibitor tablets (Roche). Insoluble material was sedimented by centrifugation and protein concentration in the cleared lysates was measured using Bradford reagent (Bio-Rad). Samples were run on 14% or 15% SDS-PAGE and transferred onto nitrocellulose filters. The filters were incubated in blocking buffer overnight (5% non-fat dried milk in TBS +0.1% Tween-20) and then incubated in primary antibodies diluted in blocking buffer for 1 hour. Antibodies used were rabbit anti-CDNF [3], rabbit anti-MANF [21], rabbit anti-DmManf [4], anti-V5 (Invitrogen), anti-α-tubulin (DM1A, Sigma) and anti-acetylated α-tubulin (6-11B-1, Sigma; Figures 2A, 2D and S1F). Secondary antibody incubations and enhanced chemiluminescence detection were performed according to standard protocols. Molecular weights were approximated by using the science gateway Protein Molecular Weight calculator (http://www.sciencegateway.org/tools/proteinmw.htm). For infrared imaging, blots were incubated with IRDye secondary antibodies (Li-Cor, Lincoln, NE, USA) according to the manufacturer’s instructions. Blots were visualized by the Odyssey infrared imager (Li-Cor) and band intensities were estimated using the ImageJ program [31].

### Immunohistochemistry, Confocal Microscopy and Image Analysis

Third instar larvae were fixed and immunostained with rabbit anti-DmManf [4] according to standard protocols. Samples were analyzed with TCS SP5 AOBS (Leica Microsystems) equipped with HCX PL APO 20x/0.7 Imm Corr (glycerol) or HCX APO 63x/1.30 Corr (glycerol) CS 21 objectives. ImageJ 1.43u was used to create representative images of confocal stacks [31]. AutoQuant X3 (MediaCybernetics) was used in deconvolution and Imaris 7.6.0 (Bitplane AG) in quantification of ER localized and total cellular DmManf.

### Constructs used in Transient Transfection

DmManf-Δss cDNA was subcloned from the corresponding construct in pBlueScript (Table S1) to the pCR3.1 vector as a *Pst*I–*Xho*I fragment. V5-DmManf-pMT, V5-DmManf-Δss-pMT, V5-DmManf-ΔRSEL-pMT, V5-DmManf-C129S-pMT, V5-DmManf-NMG1-pMT, and V5-DmManf-NMG2-pMT were cloned as follows: V5-tag was added to the corresponding constructs in pBlueScript (Table S1) by inverse PCR mutagenesis with forward primer V5Dm.F and reverse primers V5-Dm-5atg.R (DmManf-Δss) or V5-Dm-ss.R (other constructs) and subcloned as *Eco*RI-*Xho*I fragments to pMT(A) (Invitrogen).

### Transfection of S2 and CHO Cells

Schneider 2 (S2) cells were cultured in M3-BPYE medium (Shields and Sang M3, 0.5 g/l KHCO_3_, 1.0 g/l yeast extract, 2.5 g/l bactopeptone and 10% fetal bovine serum, pH 6.6) at +25°C. Transfections were performed using Fugene HD reagent (Roche). Expression from the metallothionein promoter of pMT was induced with 600 µM CuSO_4_. Media and cells were collected 3 days post-transfection. Chinese hamster ovary (CHO) cells were cultured in DMEM, 10% fetal bovine serum and antibiotics. The cells were plated in 12-well plates and on the following day transfected with the expression plasmids using Fugene HD reagent (Roche). After 3 days media and cells were collected.

### Dopamine Concentration Measurements

Larvae were collected from apple juice plates 50–55 h after egg laying in 0.5–3.2 mg samples and homogenized with an ultrasonic processor. Analysis was done with high-performance liquid chromatography (HPLC) using a Gemini C18 3 µm, 4.6×75 mm, column (Phenomenex). A 12-channel ESA CoulArray Electrode Array Detector system and CoulArray for Windows software (ESA inc.) was used for quantification of dopamine [32]. Dopamine was identified by both exact retention time and characteristic electrochemical properties.

### RT-PCR

Total RNA was extracted from 50 1^st^ instar larvae (DmManf samples) or 5 adults (HsMANF and HsCDNF samples) by RNeasy Mini Kit (Qiagene) and treated with RQ1 RNase-Free DNase (Promega). M-MLV Reverse Transcriptase (Promega) was used in cDNA synthesis and GoTaq® Hot Start Polymerase in PCR reaction. Primers and expected sizes of PCR products are listed in Table S3.

### Microinjection of SCG Neurons

DmManf and C-DmManf-Δss cDNA:s were subcloned from corresponding constructs in pBlueScript (Table S1) to the pCR3.1 vector as *Pst*I-*Xho*I fragments. SCG neurons were prepared from neonatal mice anesthetized by hypothermia and sacrificed by decapitation. All procedures for animal use were approved by the University of Helsinki Laboratory Animal Centre (Protocol number KEK11-020). Neurons were cultured with NGF (Promega) for 5–6 days and microinjected with the plasmids encoding for the respective DmManf constructs or the empty vector (negative control) together with the reporter plasmid of EGFP. Immediately after microinjection, the neurons were treated with 30 µM of etoposide (Sigma-Aldrich) in the presence of NGF. Living EGFP-expressing neurons were counted three days later in a “blind” manner and expressed as percent of original injected neurons. The NGF-maintained untreated neurons show the absence of cell death without etoposide. Average ± SEM of 2–5 independent experiments is shown. Data from each experimental group was compared with control plasmid pCR3.1-injected neurons (vector).

### Statistical Analysis

The means were compared by one-way ANOVA and *post hoc* Dunnett’s test (SCG neuron analysis) or by Student’s *t*-test (dopamine concentration analysis and ER-localised DmManf analysis). The null hypothesis was rejected at p<0.05. Statistical analysis was performed by using GraphPad InStat 3 program (GraphPad Software, Inc.; SCG neuron analysis) or Microsoft® Excel Analysis ToolPak (Microsoft® Office Professional Plus 2010; other analyses).

## Supporting Information

Figure S1 **Additional information of mutated DmManf constructs.** A) Schematic presentation of combinations of DmManf N- and C-terminal domain constructs (with or without secretion signal peptide). Colours are according to Figure 1C. Results of each rescue experiment are presented in Table 1. B) Expression analysis of the DmManf-Δss construct in wild type background by Western blotting from 3^rd^ instar larvae. Expression level of the DmManf-Δss was increased by two copies of construct and by two copies of *da*-GAL4 driver and compared to that of the DmManf construct and endogenous expression of DmManf in wild type. C) Western blot analysis of transiently transfected Chinese hamster ovary cells reveals that the DmManf-Δss is not secreted while DmManf and DmManf-ΔRSEL are. Etoposide treatment does not cause leakage of DmManf-Δss from cells into the medium. D) *In vivo* expression analysis of DmManf (b, e and h) and DmManf-Δss (c, f and i) constructs in wing discs of 3^rd^ instar larvae overexpressed by *salm*-GAL4. Overexpression of lacZ (a, d and g) was used as a control for endogenous DmManf. Transgene expression was detected as upregulation of DmManf immunoreactivity (red; d–f) in *salm*-GAL4 expression pattern marked by nuclear GFP (GFP.nls in green; a–c). White arrows (h and i) indicate the secreted DmManf. E) Rescue of *DmManf^Δ96^* mutant pupal lethality by wild type or mutated DmManf, HsMANF or HsCDNF. Wild type flies were used as control. Average ± SD. F) Expression level of *da*-GAL4 and 69B-GAL4 in 3^rd^ instar larvae by Western blot analysis. Endogenous DmManf expression in wild type background was compared to transgenic DmManf (L5) expressed by *da*-GAL4 or 69B-GAL4 in homozygous *DmManf^Δ96^* mutant background. G) Western blot analysis of cell lysates and medium from Schneider 2 cells transiently transfected with wild type and mutated V5-tagged DmManf-pMT constructs. Band intensities were normalised to DmManf. In Western blotting analyses, alpha-tubulin was used as loading control.
(TIF)

Figure S2 **Extra N-terminal amino acid residues disrupt HsCDNF functionality** ***in vivo*** **.** A) Schematic presentation of HsCDNF, HsCDNF-6N9C, HsCDNF-6N, HsCDNF-9C, N-HsCDNF, C-HsCDNF and C-HsCDNF-Δss constructs, colours according to Figure 1B. Since either the N- or C-terminal extra residues of HsCDNF-6N9C, or both, could be responsible for the loss of its functionality, we designed two HsCDNF transgenes, one with extra N-terminal residues (SLLTQG; HsCDNF-6N) and the other with extra C-terminal residues (LEGTSRGSL; HsCDNF-9C). Gray boxes indicate the additional N- and C-terminal amino acids in HsCDNF-6N9C, HsCDNF-6N and HsCDNF-9C constructs. Honeybee melittin was used as a secretion signal. B) Similarly to HsCDNF, HsCDNF-6N9C is expressed and secreted from transiently transfected Schneider 2 cells. Thus, the negative rescue result by HsCDNF-6N9C was likely not due to an expression or secretion defect. C) HsCDNF-6N9C fails to rescue *DmManf^Δ96^* mutant lethality while HsCDNF-9C fully rescues, similar to the HsCDNF construct. HsCDNF-6N shows only mild rescue of *DmManf^Δ96^* mutant lethality. This suggested that the extra six N-terminal residues in the original HsCDNF-6N9C construct were responsible for the loss of functionality. Constructs were ubiquitously expressed by *da*-GAL4. L, independent insertions of the constructs.
(TIF)

Figure S3 **Independent N- and C-terminal domains of HsMANF and HsCDNF fail to rescue DmManf mutant lethality.** A) Schematic presentation of HsMANF, N-HsMANF, C-HsMANF and C-HsMANF-Δss constructs. N- and C-terminal domain constructs failed to rescue *DmManf* mutant lethality (Table 3). Colours are according to Figure 1B. B–C) Protein expression of HsMANF (B) and HsCDNF (C) constructs was verified by Western blotting from 3^rd^ instar larvae. Constructs were ubiquitously expressed by *tub*-GAL4 driver in the wild type or heterozygous (red typing) *DmManf* mutant backgrounds. Coloured boxes under the blot indicate the domains of the construct corresponding to Figure S2A and Figure S3A. Calculated molecular weights of full length proteins, N- and C-terminal domains are presented next to Western blot images. L, independent insertions of the constructs. Alpha-tubulin was used as a loading control. D–E) Transcription from N- and C-terminal domain constructs of HsMANF (D) and HsCDNF (E) was verified by RT-PCR from adult flies. Constructs were expressed by *tub*-GAL4 in wild type background. Wild type HsMANF and HsCDNF constructs were used as positive controls, wild type flies as negative controls. Coloured boxes indicate the domains of the construct corresponding to Figure S2A and Figure S3A.
(TIF)

Table S1 **List of constructs used in generation of transgenic flies.** Cloning primers are presented in Table S2. Cloning details for 1–8 are presented in Materials and Methods. del, deletion; ins, insertion; ss, secretion signal.
(PDF)

Table S2 **List of primers used in cloning of constructs for generation of transgenic flies and in RT-PCR.**
(PDF)

Table S3 **Primer pairs used in RT-PCR to detect mRNA expression.**
(PDF)

## References

[1] Lindholm P, Saarma M (2010) Novel CDNF/MANF family of neurotrophic factors. Dev Neurobiol 70: 360–371. 20186704 10.1002/dneu.20760

[2] Petrova PS, Raibekas A, Pevsner J, Vigo N, Anafi M, et al (2003) MANF: A new mesencephalic, astrocyte-derived neurotrophic factor with selectivity for dopaminergic neurons. J Mol Neurosci 20: 173–187. 12794311 10.1385/jmn:20:2:173

[3] Lindholm P, Voutilainen MH, Laurén J, Peränen J, Leppänen V-M, et al (2007) Novel neurotrophic factor CDNF protects and rescues midbrain dopamine neurons in vivo. Nature 448: 73–77. 17611540 10.1038/nature05957

[4] Palgi M, Lindström R, Peränen J, Piepponen TP, Saarma M, et al (2009) Evidence that DmMANF is an invertebrate neurotrophic factor supporting dopaminergic neurons. Proc Natl Acad Sci U S A 106: 2429–2434. 19164766 10.1073/pnas.0810996106 PMC2650173

[5] Voutilainen MH, Bäck S, Pörsti E, Toppinen L, Lindgren L, et al (2009) Mesencephalic astrocyte-derived neurotrophic factor is neurorestorative in rat model of Parkinson’s disease. J Neurosci 29: 9651–9659. 19641128 10.1523/JNEUROSCI.0833-09.2009 PMC6666534

[6] Airavaara M, Harvey BK, Voutilainen MH, Shen H, Chou J, et al (2012) CDNF protects the nigrostriatal dopamine system and promotes recovery after MPTP treatment in mice. Cell Transplant 21: 1213–1223. 21943517 10.3727/096368911X600948 PMC3753365

[7] Airavaara M, Chiocco MJ, Howard DB, Zuchowski KL, Peränen J, et al (2010) Widespread cortical expression of MANF by AAV serotype 7: Localization and protection against ischemic brain injury. Exp Neurol 225: 104–113. 20685313 10.1016/j.expneurol.2010.05.020 PMC2925275

[8] Glembotski CC, Thuerauf DJ, Huang C, Vekich JA, Gottlieb RA, et al (2012) Mesencephalic astrocyte-derived neurotrophic factor protects the heart from ischemic damage and is selectively secreted upon sarco/endoplasmic reticulum calcium depletion. J Biol Chem 287: 25893–25904. 22637475 10.1074/jbc.M112.356345 PMC3406674

[9] Mizobuchi N, Hoseki J, Kubota H, Toyokuni S, Nozaki J-I, et al (2007) ARMET is a soluble ER protein induced by the unfolded protein response via ERSE-II element. Cell Struct Funct 32: 41–50. 17507765 10.1247/csf.07001

[10] Apostolou A, Shen Y, Liang Y, Luo J, Fang S (2008) Armet, a UPR-upregulated protein, inhibits cell proliferation and ER stress-induced cell death. Exp Cell Res 314: 2454–2467. 18561914 10.1016/j.yexcr.2008.05.001 PMC6719340

[11] Tadimalla A, Belmont PJ, Thuerauf DJ, Glassy MS, Martindale JJ, et al (2008) Mesencephalic astrocyte-derived neurotrophic factor is an ischemia-inducible secreted endoplasmic reticulum stress response protein in the heart. Circ Res 103: 1249–1258. 18927462 10.1161/CIRCRESAHA.108.180679 PMC2746824

[12] Palgi M, Greco D, Lindström R, Auvinen P, Heino TI (2012) Gene expression analysis of Drosophila Manf mutants reveals perturbations in membrane traffic and major metabolic changes. BMC Genomics 134. 10.1186/1471-2164-13-134 PMC3364883 22494833

[13] Ryoo HD, Steller H (2007) Unfolded protein response in Drosophila: Why another model can make it fly. Cell Cycle 6: 830–835. 17387279 10.4161/cc.6.7.4064

[14] Matus S, Glimcher LH, Hetz C (2011) Protein folding stress in neurodegenerative diseases: A glimpse into the ER. Curr Opin Cell Biol 23: 239–252. 21288706 10.1016/j.ceb.2011.01.003

[15] Parkash V, Lindholm P, Peränen J, Kalkkinen N, Oksanen E, et al (2009) The structure of the conserved neurotrophic factors MANF and CDNF explains why they are bifunctional. Protein Eng Des Sel 22: 233–241. 19258449 10.1093/protein/gzn080

[16] Hoseki J, Sasakawa H, Yamaguchi Y, Maeda M, Kubota H, et al (2010) Solution structure and dynamics of mouse ARMET. FEBS Lett 584: 1536–1542. 20214902 10.1016/j.febslet.2010.03.008

[17] Hellman M, Arumäe U, Yu L-Y, Lindholm P, Peränen J, et al (2011) Mesencephalic astrocyte-derived neurotrophic factor (MANF) has a unique mechanism to rescue apoptotic neurons. J Biol Chem 286: 2675–2680. 21047780 10.1074/jbc.M110.146738 PMC3024763

[18] Bruhn H (2005) A short guided tour through functional and structural features of saposin-like proteins. Biochem J 389: 249–257. 15992358 10.1042/BJ20050051 PMC1175101

[19] Sawada M, Hayes P, Matsuyama S (2003) Cytoprotective membrane-permeable peptides designed from the Bax-binding domain of Ku70. Nature Cell Biol 5: 352–357. 12652309 10.1038/ncb955

[20] Ellgaard L, Ruddock LW (2005) The human protein disulphide isomerase family: Substrate interactions and functional properties. EMBO Rep 6: 28–32. 15643448 10.1038/sj.embor.7400311 PMC1299221

[21] Lindholm P, Peränen J, Andressoo J-O, Kalkkinen N, Kokaia Z, et al (2008) MANF is widely expressed in mammalian tissues and differently regulated after ischemic and epileptic insults in rodent brain. Mol Cell Neurosci 39: 356–371. 18718866 10.1016/j.mcn.2008.07.016

[22] Oh-Hashi K, Tanaka K, Koga H, Hirata Y, Kiuchi K (2012) Intracellular trafficking and secretion of mouse mesencephalic astrocyte-derived neurotrophic factor. Mol Cell Biochem 363: 35–41. 22120531 10.1007/s11010-011-1155-0

[23] McQuilton P, St Pierre SE, Thurmond J, Gelbart W, Brown N, et al (2012) FlyBase 101 - the basics of navigating FlyBase. Nucleic Acids Res 40: D706–D714. 22127867 10.1093/nar/gkr1030 PMC3245098

[24] Birman S, Morgan B, Anzivino M, Hirsh J (1994) A novel and major isoform of tyrosine hydroxylase in Drosophila is generated by alternative RNA processing. J Biol Chem 269: 26559–26567. 7929381

[25] Brand AH, Perrimon N (1993) Targeted gene expression as a means of altering cell fates and generating dominant phenotypes. Development 118: 401–415. 8223268 10.1242/dev.118.2.401

[26] Shiga Y, Tanaka-Matakatsu M, Hayashi S (1996) A nuclear GFP/β-galactosidase fusion protein as a marker for morphogenesis in living Drosophila. Dev Growth Differ 38: 99–106.

[27] LaJeunesse DR, Buckner SM, Lake J, Na C, Pirt A, et al (2004) Three new Drosophila markers of intracellular membranes. BioTechniques 36: 784–790. 15152597 10.2144/04365ST01

[28] Wodarz A, Hinz U, Engelbert M, Knust E (1995) Expression of crumbs confers apical character on plasma membrane domains of ectodermal epithelia of Drosophila. Cell 82: 67–76. 7606787 10.1016/0092-8674(95)90053-5

[29] Lee T, Luo L (1999) Mosaic analysis with a repressible neurotechnique cell marker for studies of gene function in neuronal morphogenesis. Neuron 22: 451–461. 10197526 10.1016/s0896-6273(00)80701-1

[30] Hinz U, Giebel B, Campos-Ortega JA (1994) The basic-helix-loop-helix domain of Drosophila lethal of scute protein is sufficient for proneural function and activates neurogenic genes. Cell 76: 77–87. 8287481 10.1016/0092-8674(94)90174-0

[31] Abràmoff MD, Magalhães PJ, Ram SJ (2004) Image processing with ImageJ. Biophoton Int 11: 36–41.

[32] Airavaara M, Mijatovic J, Vihavainen T, Piepponen TP, Saarma M, et al (2006) In heterozygous GDNF knockout mice the response of striatal dopaminergic system to acute morphine is altered. Synapse 59: 321–329. 16437537 10.1002/syn.20245

