# Supplementary figures and images for: Characterization of the Structural and Functional Determinants of MANF/CDNF in Drosophila In Vivo Model
Source: PLoS One. 2013 Sep 3;8(9):e73928. doi: 10.1371/journal.pone.0073928 (PMC3760817; doi:10.1371/journal.pone.0073928)

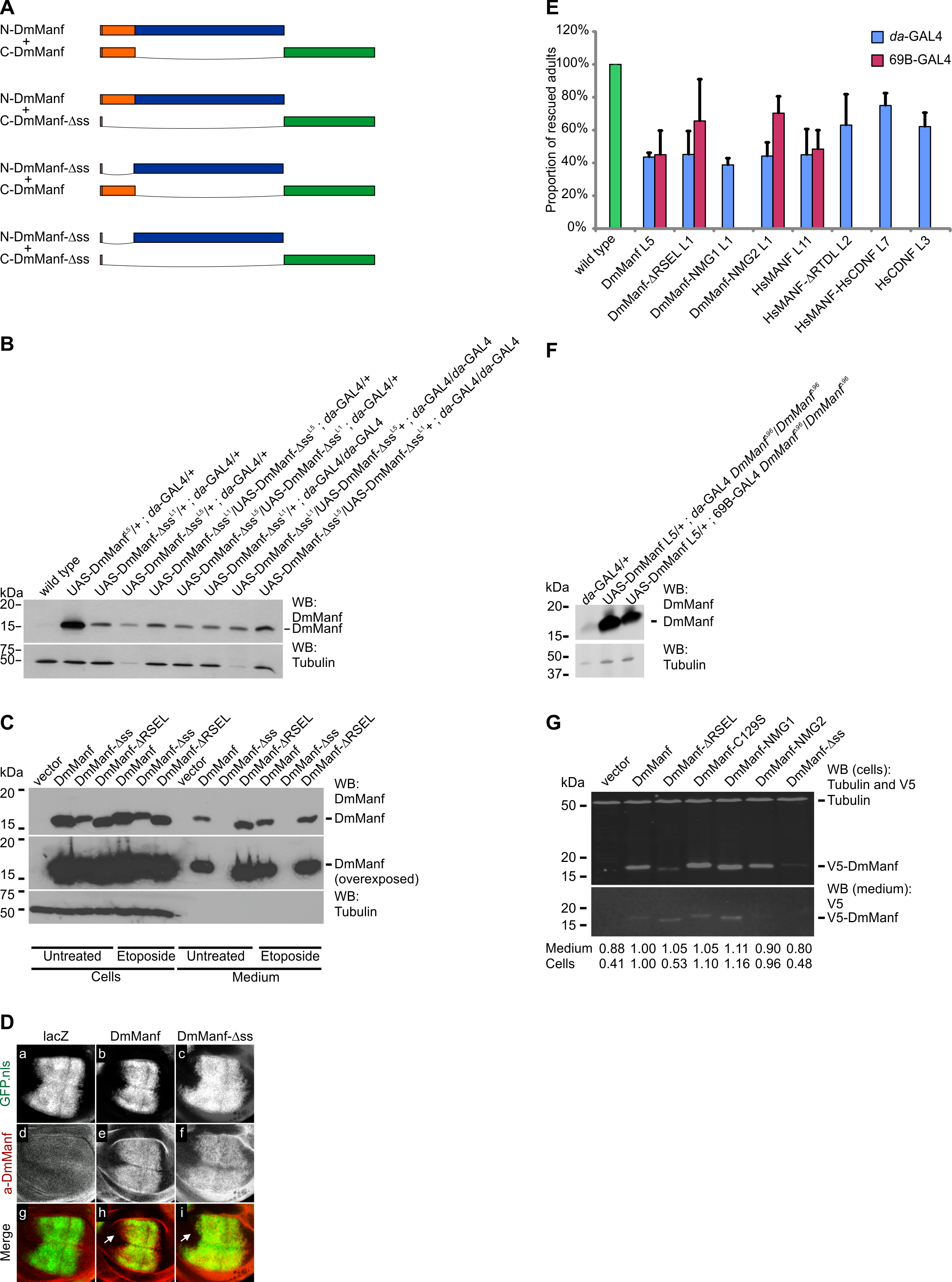

Supplement: Figure S1 — Additional information of mutated DmManf constructs. A) Schematic presentation of combinations of DmManf N- and C-terminal domain constructs (with or without secretion signal peptide). Colours are according to Figure 1C. Results of each rescue experiment are presented in Table 1. B) Expression analysis of the DmManf-Δss construct in wild type background by Western blotting from 3rd instar larvae. Expression level of the DmManf-Δss was increased by two copies of construct and by two copies of da-GAL4 driver and compared to that of the DmManf construct and endogenous expression of DmManf in wild type. C) Western blot analysis of transiently transfected Chinese hamster ovary cells reveals that the DmManf-Δss is not secreted while DmManf and DmManf-ΔRSEL are. Etoposide treatment does not cause leakage of DmManf-Δss from cells into the medium. D) In vivo expression analysis of DmManf (b, e and h) and DmManf-Δss (c, f and i) constructs in wing discs of 3rd instar larvae overexpressed by salm-GAL4. Overexpression of lacZ (a, d and g) was used as a control for endogenous DmManf. Transgene expression was detected as upregulation of DmManf immunoreactivity (red; d–f) in salm-GAL4 expression pattern marked by nuclear GFP (GFP.nls in green; a–c). White arrows (h and i) indicate the secreted DmManf. E) Rescue of DmManfΔ96 mutant pupal lethality by wild type or mutated DmManf, HsMANF or HsCDNF. Wild type flies were used as control. Average ± SD. F) Expression level of da-GAL4 and 69B-GAL4 in 3rd instar larvae by Western blot analysis. Endogenous DmManf expression in wild type background was compared to transgenic DmManf (L5) expressed by da-GAL4 or 69B-GAL4 in homozygous DmManfΔ96 mutant background. G) Western blot analysis of cell lysates and medium from Schneider 2 cells transiently transfected with wild type and mutated V5-tagged DmManf-pMT constructs. Band intensities were normalised to DmManf. In Western blotting analyses, alpha-tubulin was used as loading control. (TIF) [file pone.0073928.s001.tif]

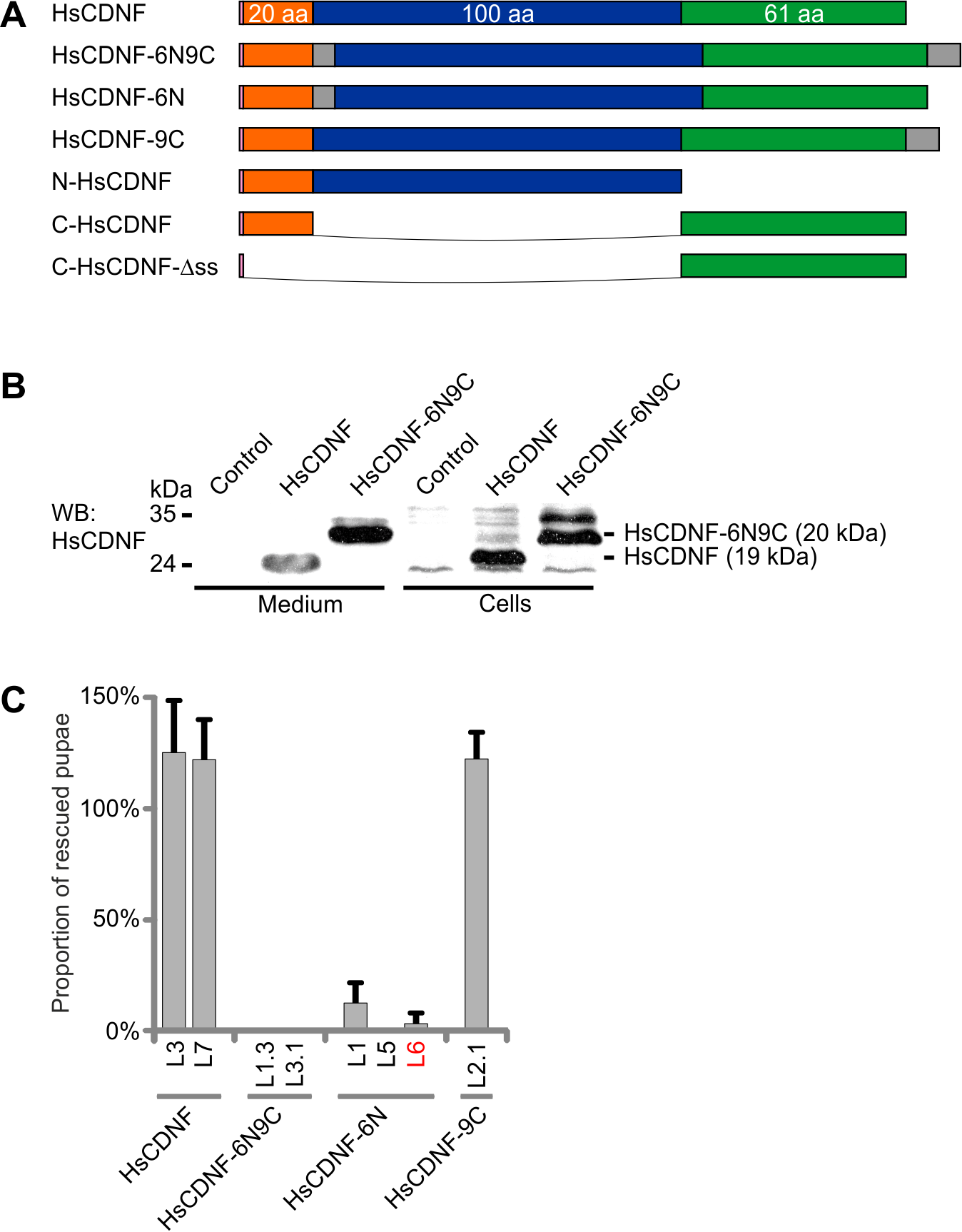

Supplement: Figure S2 — Extra N-terminal amino acid residues disrupt HsCDNF functionality in vivo . A) Schematic presentation of HsCDNF, HsCDNF-6N9C, HsCDNF-6N, HsCDNF-9C, N-HsCDNF, C-HsCDNF and C-HsCDNF-Δss constructs, colours according to Figure 1B. Since either the N- or C-terminal extra residues of HsCDNF-6N9C, or both, could be responsible for the loss of its functionality, we designed two HsCDNF transgenes, one with extra N-terminal residues (SLLTQG; HsCDNF-6N) and the other with extra C-terminal residues (LEGTSRGSL; HsCDNF-9C). Gray boxes indicate the additional N- and C-terminal amino acids in HsCDNF-6N9C, HsCDNF-6N and HsCDNF-9C constructs. Honeybee melittin was used as a secretion signal. B) Similarly to HsCDNF, HsCDNF-6N9C is expressed and secreted from transiently transfected Schneider 2 cells. Thus, the negative rescue result by HsCDNF-6N9C was likely not due to an expression or secretion defect. C) HsCDNF-6N9C fails to rescue DmManfΔ96 mutant lethality while HsCDNF-9C fully rescues, similar to the HsCDNF construct. HsCDNF-6N shows only mild rescue of DmManfΔ96 mutant lethality. This suggested that the extra six N-terminal residues in the original HsCDNF-6N9C construct were responsible for the loss of functionality. Constructs were ubiquitously expressed by da-GAL4. L, independent insertions of the constructs. (TIF) [file pone.0073928.s002.tif]

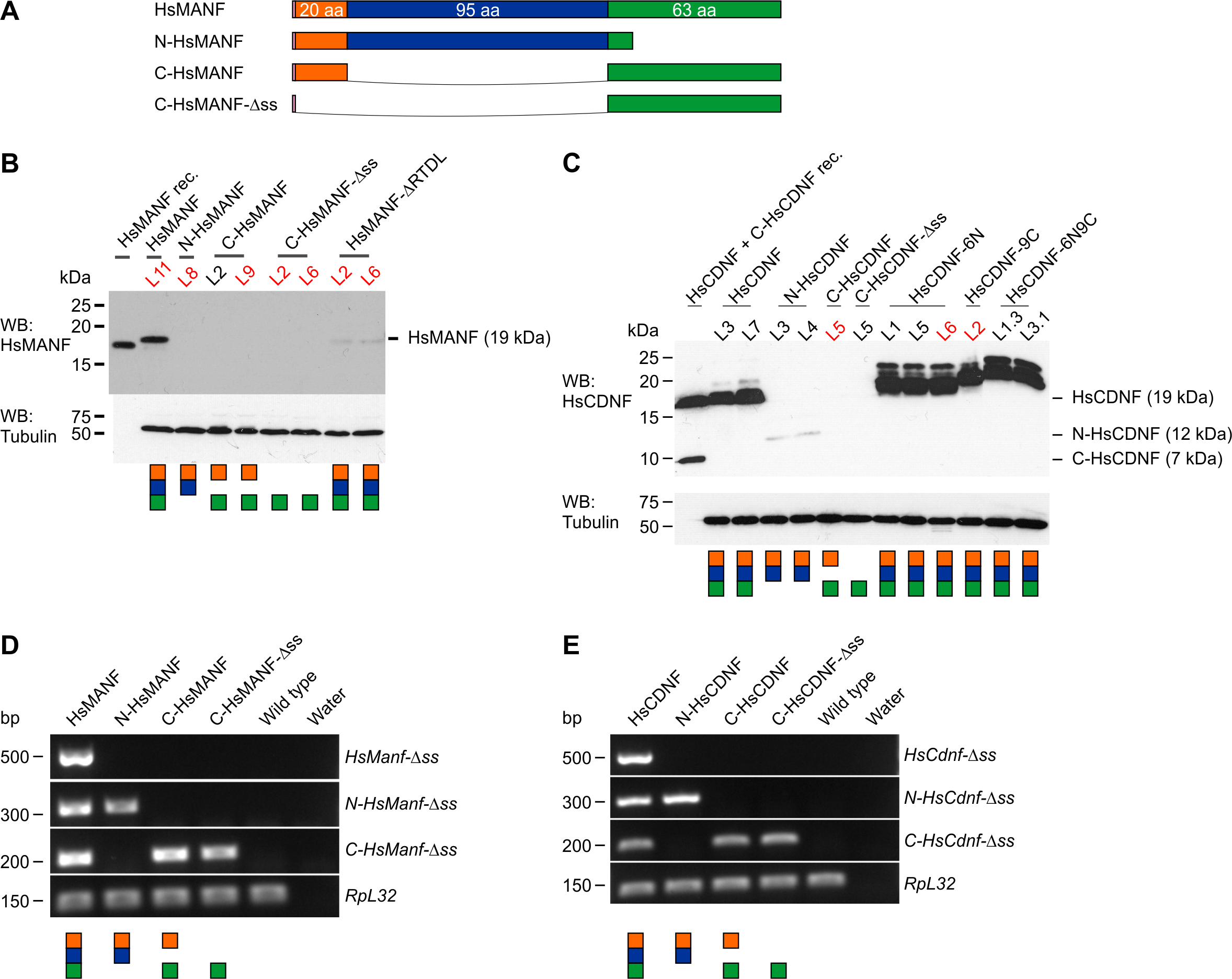

Supplement: Figure S3 — Independent N- and C-terminal domains of HsMANF and HsCDNF fail to rescue DmManf mutant lethality. A) Schematic presentation of HsMANF, N-HsMANF, C-HsMANF and C-HsMANF-Δss constructs. N- and C-terminal domain constructs failed to rescue DmManf mutant lethality (Table 3). Colours are according to Figure 1B. B–C) Protein expression of HsMANF (B) and HsCDNF (C) constructs was verified by Western blotting from 3rd instar larvae. Constructs were ubiquitously expressed by tub-GAL4 driver in the wild type or heterozygous (red typing) DmManf mutant backgrounds. Coloured boxes under the blot indicate the domains of the construct corresponding to Figure S2A and Figure S3A. Calculated molecular weights of full length proteins, N- and C-terminal domains are presented next to Western blot images. L, independent insertions of the constructs. Alpha-tubulin was used as a loading control. D–E) Transcription from N- and C-terminal domain constructs of HsMANF (D) and HsCDNF (E) was verified by RT-PCR from adult flies. Constructs were expressed by tub-GAL4 in wild type background. Wild type HsMANF and HsCDNF constructs were used as positive controls, wild type flies as negative controls. Coloured boxes indicate the domains of the construct corresponding to Figure S2A and Figure S3A. (TIF) [file pone.0073928.s003.tif]
