# Supplementary material for: Characterization of the Structural and Functional Determinants of MANF/CDNF in Drosophila In Vivo Model
Source: PLoS One. 2013 Sep 3;8(9):e73928. doi: 10.1371/journal.pone.0073928 (PMC3760817; doi:10.1371/journal.pone.0073928)
Supplement: Table S1 — List of constructs used in generation of transgenic flies. Cloning primers are presented in Table S2. Cloning details for 1–8 are presented in Materials and Methods. del, deletion; ins, insertion; ss, secretion signal. (PDF) [file pone.0073928.s004.pdf]

Table S1: List of constructs used in generation of transgenic flies.

Transgene	Modification	Template in Bluescript/pCR3.1	Forward primer	Reverse primer	to pUAST
DmMANF-Δss	del(ss)	DmMANF-BlueScript	DmMANF_nosec.fwd	DmMANF-5atg.rev	*Eco*RI-*Xho*I
N-DmMANF	del(C-term)	DmMANF-BlueScript	DmMANF-3stop.fwd	DmMANF-Nterm.rev	*Eco*RI-*Xho*I
DmMANF-Δss	del(C-term), del(ss)	N-DmMANF-BlueScript	DmMANF_nosec.fwd	DmMANF-5atg.rev	*Eco*RI-*Xho*I
C-DmMANF	del(N-term)	DmMANF-BlueScript	DmMANF-Cterm.fwd	DmMANF-secr.rev	*Eco*RI-*Xho*I
C-DmMANF-Δss	del(N-term), del(ss)	DmMANF-BlueScript	DmMANF-Cterm.fwd	DmMANF-5atg.rev	*Eco*RI-*Xho*I
DmMANF-ΔRSEL	del(RSEL)	DmMANF-BlueScript	DmMANF-3stop.fwd	DmMANF_delRSEL.rev	*Eco*RI-*Xho*I
DmMANF-C129S	C129S	DmMANF-BlueScript	DmMANF_C129S.fwd	DmMANF_int.rev	*Eco*RI-*Xho*I
DmMANF-NMG1	K79A, K83A, K86A	DmMANF-BlueScript	Dm_K86A.fwd	Dm_K79A_K83A.rev	*Eco*RI-*Xho*I
DmMANF-NMG2	K43A, K45A, R95A	DmMANF-K95A-BlueScript (1)	DmMANF_K45A.fwd	DmMANF_K43A.rev	*Eco*RI-*Xho*I
HsMANF	-	(2)			*Eco*RI-*Xho*I
N-HsMANF	del(C-term)	HsMANF-pCR3.1			*Eco*RI-*Xho*I
C-HsMANF	del(N-term)	HsMANF-pCR3.1	HsMANF-Cterm.fwd	HsMANF-mel.rev	*Eco*RI-*Xho*I
C-HsMANF-Δss	del(C-term), del(ss)	HsMANF-pCR3.1	HsMANF-Cterm.fwd	HsMANF-5atg-pCR31.rev	*Eco*RI-*Xho*I
HsMANF-ΔRTDL	del(RTDL)	HsMANF-pCR3.1	HsMANF-3stop.fwd	hMANFdel(RTDL)_R	*Eco*RI-*Xho*I
HsMANF-HsCDNF	del(C-term), ins(CDNF C-term)	(3)			*Eco*RI-*Xho*I
HsCDNF	-	(4)			
N-HsCDNF	del(C-term)	HsCDNF-BlueScript (5)	HsCDNF-3stop.fwd	HsCDNF-Nterm.rev	*Not*I-*Xho*I
C-HsCDNF	del(N-term)	HsCDNF-BlueScript (5)	HsCDNF-Cterm.fwd	HsMANF-mel.rev	*Not*I-*Xho*I
C-HsCDNF-Δss	del(N-term), del(ss)	(6)			*Bgl*II-*Xho*I
HsCDNF-6N	ins(6N)	(7)			*Not*I-*Xho*I
HsCDNF-9C	ins(9C)	HsCDNF-6N9C-del(stop)-BlueScript (8)	CDNF_nosec.fwd	CDNF_melGML.rev	*Not*I-*Xho*I

Cloning primers are presented in Table S2. Cloning details for 1–8 are presented in Materials and Methods. del, deletion; ins, insertion; ss, secretion signal.
