# Supplementary material for: Characterization of the Structural and Functional Determinants of MANF/CDNF in Drosophila In Vivo Model
Source: PLoS One. 2013 Sep 3;8(9):e73928. doi: 10.1371/journal.pone.0073928 (PMC3760817; doi:10.1371/journal.pone.0073928)
Supplement: Table S2 — List of primers used in cloning of constructs for generation of transgenic flies and in RT-PCR. (PDF) [file pone.0073928.s005.pdf]

Table S2: List of primers used in cloning of constructs for generation of transgenic flies and in RT-PCR.

Primer	Sequence (5' -> 3')
DmMANF-5atg.rev	CATTTTGTTATTACTTCGACTGTGCTTGAGCG
DmMANF-3stop.fwd	TAGAGCGCAGTCAGTTACTTGTGTAG
DmMANF-Nterm.rev	AAGGTCGCAGATTTGTGCGTCC
DmMANF-Cterm.fwd	CGCTATGAGAAACAAATCGATCTGAACAG
DmMANF-secr.rev	GGCCAGCGACGTTTGGGCCAACG
DmMANF_nosec.fwd	CTGAAAGAGGAGGACTGCGAAGTTTG
DmMANF_delRSEL.rev	CGAGTACTTGGGCTTCAGCTCC
DmMANF_C129S.fwd	GTGACGGTAGCCTGGAGAAGG
DmMANF_int.rev	AGCTCTCGTCCCAGTCGTTGAG
Dm_K79A_K83A.rev	CGCCTCGCAGATCGCCTCAGCTG
Dm_K86A.fwd	CTGAAGGCGAAGGACGCACAAATCTG
DmMANF_K43A.rev	GTTTGCCTGCGCTTTGCAGAACTTTTTG
DmMANF_K45A.fwd	GCGGAACACAGATTCTGTTACTACCTCG
DmMANF_R95A.fwd	GCCTATGAGAAACAAATCGATCTGAACAG
HsMANF-Cterm.fwd	AAGTATGACAAGCAGATCGACCTGAG
HsMANF-mel.rev	GGCATAGATGTAAGAAATGTATACGACC
HsMANF-5atg-pCR31.rev	CATGGTAGCTTTAAATTCGAATTCCACCAC
HsMANF-3stop.fwd	TAGCTCGAGTCTAGAGGGCCCGTTTAA
hMANFdel(RTDL)_R	TGCACTGGCTGCCTTGGGGGCA
NotI-CDNF-pUAST.fwd	GAACGCGGCCGCGGGAATTGGGAATTCGAATTTAAAG
CDNF-pUAST-XhoI.rev	CCAGCTCGAGGGATATCTGCAGAATTCGCCCTTG
NotI_CDNF_old_pUAST.fwd	CTGAGCGGCCGCTTGGGAATTCGTTAACAG
CDNF_old_pUAST_XbaI.rev	ACACTCTAGAAGTAAGGTTCCTTCAC
CDNF_nosec.fwd	CAGGAGGCCGGGGGGCGG
CDNF_melGML.rev	TAGCATGCCGGCATAGATGTAAGAAATG
HsCDNF-3stop.fwd	TGATCAAGGGCGAATTCTGCAGATATCC
HsCDNF-Nterm.rev	CAGCTCACAGATCTGGCTATCCAACTTC
HsCDNF-Cterm.fwd	AAATATGAAAAAACACTGGACTTGGCATCAG
BglII-M-HsCDNF-Cterm.fwd	AGCCAGATCTGTGAGATGAAATATG
HsCDNF-TOPO-XhoI.rev	TAGATGCATGCTCGAGCGG
HsMANF_nosec.fwd	ATAAGGATCCATGCTGCGGCCGGGCGACTGCGA
HsMANF_stop.rev	CTACAAATCGGTCCGTGCACTGGCTG
HsMANF-Nterm.rev	TAAACGTCGACTTAAAGCTCACATATCTGGCTGTCCTT
HsCDNF-delstop.rev	GAGCTCTGTTTTGGGGTGTGTC
V5-Dm-5atg.R	AGGGTTAGGGATAGGCTTACCCATTTTGTTATTACTTCGACTGTG CTTGAG
V5-Dm-ss.R	AGGGTTAGGGATAGGCTTACCGGCCAGCGACGTTTGGGCCAAC
V5Dm.F	CTCCTCGGTCTCGATTCTACGCTGAAAGAGGAGGACTGCGAAG
