# Supplementary material for: Characterization of the Structural and Functional Determinants of MANF/CDNF in Drosophila In Vivo Model
Source: PLoS One. 2013 Sep 3;8(9):e73928. doi: 10.1371/journal.pone.0073928 (PMC3760817; doi:10.1371/journal.pone.0073928)
Supplement: Table S3 — Primer pairs used in RT-PCR to detect mRNA expression. (PDF) [file pone.0073928.s006.pdf]

Table S3: Primer pairs used in RT-PCR to detect mRNA expression.

Transcript	Forward primer	Reverse primer	Expected size of PCR product (bp)
DmManf-Δss	DmMANF_nosec.fwd	DmMANF_delRSEL.rev	441
N-DmManf-Δss	DmMANF_nosec.fwd	DmMANF-Nterm.rev	282
C-DmManf-Δss	DmMANF-Cterm.fwd	DmMANF_delRSEL.rev	159
HsMANF-Δss	HsMANF_nosec.fwd	HsMANF_stop.rev	490
N-HsMANF-Δss	HsMANF_nosec.fwd	HsMANF-Nterm.rev	312
C-HsMANF-Δss	HsMANF-Cterm.fwd	HsMANF_stop.rev	192
HsCDNF-Δss	CDNF_nosec.fwd	HsCDNF-delstop.rev	483
N-HsCDNF-Δss	CDNF_nosec.fwd	HsCDNF-Nterm.rev	300
C-HsCDNF-Δss	HsCDNF-Cterm.fwd	HsCDNF-delstop.rev	183
RpL32	CGGATCGATATGCTAAGCTGT	GCGCTTGTTCGATCCGTA	122

Primers are listed in Table S2.
